# Impact of Gold Nanoparticles on the Functions of Macrophages and Dendritic Cells

**DOI:** 10.3390/cells10010096

**Published:** 2021-01-07

**Authors:** Arindam K. Dey, Alexis Gonon, Eve-Isabelle Pécheur, Mylène Pezet, Christian Villiers, Patrice N. Marche

**Affiliations:** 1 Institute for Advanced Biosciences, UMR CNRS 5309/INSERM U1209, Université Grenoble-Alpes, 38400 Grenoble, France; arindam.dey@inserm.fr (A.K.D.); alexis.gonon@gmail.com (A.G.); mylene.pezet@inserm.fr (M.P.); villiers.christ@gmail.com (C.V.); 2 Centre de Recherche en Cancérologie de Lyon, UMR CNRS 5286/INSERM U1052, Université de Lyon, 69008 Lyon, France; eve-isabelle.pecheur@inserm.fr

**Keywords:** antigen presenting cells, phagocytic cells, immunotoxicity, inflammation, immuno-metabolism

## Abstract

Gold nanoparticles (AuNPs) have demonstrated outstanding performance in many biomedical applications. Their safety is recognised; however, their effects on the immune system remain ill defined. Antigen-presenting cells (APCs) are immune cells specialised in sensing external stimulus and in capturing exogenous materials then delivering signals for the immune responses. We used primary macrophages (Ms) and dendritic cells (DCs) of mice as an APC model. Whereas AuNPs did not alter significantly Ms and DCs functions, the exposure to AuNPs affected differently Ms and DCs in their responses to subsequent stimulations. The secretion of inflammatory molecules like cytokines (IL-6, TNF-α), chemokine (MCP-1), and reactive oxygen species (ROS) were altered differently in Ms and DCs. Furthermore, the metabolic activity of Ms was affected with the increase of mitochondrial respiration and glycolysis, while only a minor effect was seen on DCs. Antigen presentation to T cells increased when DCs were exposed to AuNPs leading to stronger Th1, Th2, and Th17 responses. In conclusion, our data provide new insights into the complexity of the effects of AuNPs on the immune system. Although AuNPs may be considered as devoid of significant effect, they may induce discrete modifications on some functions that can differ among the immune cells.

## 1. Introduction

In the past few decades, nanoparticles (NPs) have been introduced in medicine for therapeutic and diagnostic applications. The surface modifications of these nanometric structures, together with the encapsulation of drugs, enable the development of innovative drug carriers or contrast agents for imaging. This field of investigation is of great interest, as NPs might enable the development of targeted therapies, increasing the diffusion and effectiveness of drugs together with facilitated administration and reduced public health costs [1].

Gold is the archetype of materials developed for medical purposes. Indeed, gold-based NPs (AuNPs) are easily synthesised and adjustable in size. Their surface can be covalently-modified and they are resistant to oxidation [2]. All these properties enable the use of AuNPs to deliver different kinds of therapeutic agents [3]. Furthermore, AuNPs have specific optical properties, and can be easily detected and imaged. Based upon these peculiarities, AuNPs have been used as photothermal agents for cancer therapy, which are capable of delivering heat upon surface plasmon oscillation excitation [4]. Despite the wide range of beneficial applications, AuNPs are thought to induce toxic side effects when inhaled or ingested. Thus, it is important to investigate their toxicity and how they can be minimised.

The immune system is specialised in the maintenance of body integrity and comprises an innate and adaptive contribution. The innate immune system is considered as the first line of defence against foreign pathogens [5]. It comprises antigen-presenting cells (APCs), such as macrophages (Ms) and dendritic cells (DCs), both found in the blood, lymphoid organs, and residing in any organs. Ms are a subset of phagocytes of the innate immune system. Their main role is to engulf and digest cellular debris and pathogens taken up from their environment by a mechanism called phagocytosis. They contribute to a rapid and non-specific defence, and are efficient against most pathogens [6]. The DCs are defined as professional APCs [7]. They act at the interface between the innate and adaptive immune systems. After phagocytosis, their main function is to process and present antigens to naïve T lymphocytes (LT), associated with class II major histocompatibility complex (MHC-II). This process may initiate the adaptive immune response. Upon activation by a foreign pathogen, DCs secrete a large range of cytokines, implicated in the activation of natural killers or the control of T cell response, for example [8]. The integrity of both these cell populations is necessary to ensure proper responses to infection; they are, thus, likely the most relevant experimental models for the study of the effects of NPs on cell fate.

Both DCs and Ms express toll-like receptors (TLR) that allow them to detect and respond to pathogen-derived molecules [9]. In response to different TLR agonists, as well as cytokines such as IL-4 and IL-13, DCs and Ms transition from a resting to an activated state through a process that involves the induction of expression of genes encoding a broad array of proteins such as cytokines, chemokines, and co-stimulatory molecules. Activation of DCs and Ms in response to particular stimulus requires support from metabolic and bioenergetic resources. Therefore, immuno-metabolism entails all activities and changes that occur in immune cells [10].

Indeed, AuNPs have been found to accumulate in Ms and DCs due to their phagocytic capacity [11], making these cells suitable to investigate the toxic effects of AuNPs. They could engulf a large volume of these particles, and in case AuNPs display high toxicity, the metabolism of Ms and DCs could be severely affected, eventually leading to the alteration of cellular functions.

Up to now, publications dealing with AuNPs toxicity demonstrated that the accumulation of these particles in cells induced low cytotoxicity [12,13,14,15]. However, the functional impact of AuNPs exposure on the immune system cells, also defined as immunotoxicity, remains poorly documented.

This paper describes the impact of AuNPs on Ms and DCs metabolism, which could lead to alteration in the respective cell function, and proposes that reprogramming of cell metabolism using AuNPs might be a novel therapeutic approach to treat inflammatory diseases.

For this reason, we analysed whether exposure to sub-toxic doses of AuNPs (10 nm and 50 nm) leads to functional alterations of these cells. We evaluated the effect of AuNPs on the following: (1) phagocytic capacity of Ms and DCs; (2) cell activation; (3) cytokine production; (4) redox profile; (5) metabolic profile, and (6) LT activation by DCs.

## 2. Materials and Methods

### 2.1. Cell Culture

The mouse Ms cell line J774.1A was obtained from the American Type Culture Collection (ATCC™). Cells were cultured in complete Dulbecco’s modified Eagle’s medium (DMEM) supplemented with 10% foetal bovine serum and 1% penicillin-streptomycin.

The bone marrow (BM) derived dendritic cells (BMDCs) were generated from BM extracted from C57BL/6 mice (Charles River, l’Arbresle, France), as described earlier [16]. Briefly, BM cells were isolated by flushing from the tibia and femurs. Erythrocytes and GR1 (Granulocytic Marker-1) positive cells were removed by magnetic cell sorting using Dynabeads (ThermoFisher, Waltham, MA, USA, cat. no.: 11047) after incubation with Ly-6G/Ly-6C (BD Pharmingen, cat. no.: 553125) and TER-119 (BD Pharmingen, cat. no.: 553672) antibodies, the remaining negatively sorted cells were isolated using Dynabeads isolation kit (ThermoFisher, cat. no.: 11047), and resuspended at 5 × 10^5^ cells/mL in complete Iscove’s modified Dulbecco’s medium (IMDM) (ThermoFisher, cat. no.: 21980065), supplemented with granulocyte-macrophage colony-stimulating factor (GM-CSF) (Peprotech, London, UK, cat. no.: 315-03), FLT-3L (Peprotech, cat. no.: 250-31L), and IL-6 (Peprotech, cat. no.: 216-16) according to Table 1. The transformation of the progenitors into fully active DCs occurred after 10 days of culture.

The bone marrow-derived macrophages (BMDMs) were generated from the BM from C57BL/6 mice, as described earlier [17]. Erythrocytes were removed by red blood cell (RBC) lysis buffer, and the remaining cells were cultured in complete DMEM medium (ThermoFisher, cat.no:61965026) supplemented), with 20% of L929 conditioned medium (source of M-CSF) for 7 days.

Ovalbumin (OVA)-specific CD4+ T cells were obtained from OT II Mice (Charles River Laboratories, l’Arbresle, France). Briefly, mouse spleen was dissociated in the Roswell Park Memorial Institute (RPMI) medium, and erythrocytes were lysed using RBC lysis buffer. The T cells were isolated by negative selection using Dynabeads^®^ Untouched™ Mouse T Cell Kit (ThermoFisher, cat. no.: 11413D) and resuspended in the culture medium of BMDCs.

### 2.2. Gold Nanoparticles (AuNPs)

BioPure Gold Nanospheres were obtained from Nanocomposix (Nanocomposix, San Diego, CA, USA, cat. no.: AUCB20). These AuNPs were of high quality particles, unagglomerated, monodispersed, extensively purified, and provided sterile and endotoxin free (<5 EU/mL). The zeta potential of these particles were +19.7 ± 0.8 mV (3 independent measurements at 25 °C) by electrophoretic light scattering (ELS) using a Zeta Sizer Nano ZS instrument (Malvern Panalytical, Malvern, UK) with 1 µg/mL AuNPs dispersion in 1 mM NaCl. The hydrodynamic diameter and polydispersity index (PDI) of these particles were measured by dynamic light scattering (DLS) with a 1 µg/mL AuNPs in complete DMEM medium. The hydrodynamic diameter was 97.01 ± 7.29 nm in DMEM (complete medium with 10% FBS) (Supplementary Table S1). The PDI was 0.45 ± 0.009.

### 2.3. Incubation with AuNPs

Cells were plated on 12, 24, or 96 well plates from Falcon^®^ or Seahorse XFe96 cell culture microplates and exposed to AuNPs at 10 and 50 µg/mL final concentrations for 24 h. This time of exposure was selected because it is actually the longest time of exposure feasible to achieve all the assay time lines without affecting cell viability. Then, the cells were washed and stimulated with lipopolysaccharide (LPS) (Sigma, Saint-Quentin Fallavier, France, cat. no.: L2654) (2 µg/mL) or IL-4 (ThermoFisher, cat. no.: 14-8041-80) (20 ng/mL) for 24 h. The impact of AuNPs on BMDMs and BMDCs was assayed for parameters such as viability, phagocytosis, activation, cytokine secretion, nitric oxide (NO) production, reactive oxygen species (ROS) production, glycolysis, or mitochondrial metabolism. A graphical representation of all the experiments is presented in Supplementary Figure S1.

### 2.4. Toxicity Assessment

Cell viability was tested by CytoTox-ONE™ Homogeneous Membrane Integrity Assay (Promega, Charbonnières-les-Bains, France, cat. no.: G7891), according to the manufacturer’s optimised protocol. Lysis solution was added to generate full lactic dehydrogenase (LDH) release corresponding to a 100% cell death and used as control. The fluorescence signal was recorded (at 560 nm and 590 nm for excitation and emission, respectively) with a CLARIOstar^®^ Microplate Reader (BMG Labtech, Ortenberg, Germany).

### 2.5. Confocal Microscopy and Transmission Electron Microscopy (TEM) Analyses

The AuNPs accumulation inside cells was analysed by the reflection of the indicated laser on their surface, using LSM510 Confocor II (Zeiss, Oberkochen, Germany). Cell membrane was visualised using FITC-conjugated cholera toxin (Sigma, cat. no.: C1655). Cell compartments were labelled with Cy3-coupled anti-early endosome antigen 1 (EEA1) (GeneTex, Irvine, CA, USA, cat. no.: GTX109638) and Alexa Fluor 647^®^ coupled anti-lysosomal associated membrane protein 1 (LAMP1) antibodies (Santa Cruz Biotechnology, Heidelberg, Germany, cat. no.: sc-8099). After incubation with AuNPs at the indicated conditions, cells washed with PBS were incubated for 1 h at room temperature in Petri dishes pre-coated with 20 mM polylysine for 24 h.

For TEM, cells were gently washed with PBS and fixed with 1% *v*/*v* glutaraldehyde in PBS for 1 h at room temperature. They were then post-fixed in 1% *v*/*v* osmium tetroxide in PBS for 1 h, and processed by ethanol dehydration, followed by embedding in epoxy resin. Observations were performed on a Philips CM120 TEM microscope operated at 80 kV.

### 2.6. Phagocytosis Assay

The AuNPs exposed J774.1A Ms were incubated with 1 µm-diameter FluoSpheres^®^ Carboxylate-Modified Microspheres (1 µm, ThermoFisher, cat. no.: F8851), at a ratio of 10 microspheres per cell for 6 h at 37 °C in a 5% CO_2_ incubator. The cells were analysed for their fluorescence on a BD Accuri™ C6 flow cytometer (BD Biosciences, Claix, France) and FCS Express V6 (De Novo Software).

### 2.7. Cell Activation

The AuNPs exposed BMDMs and BMDCs were stimulated with 2 µg/mL LPS from *E. coli*, for 24 h at 37 °C with 5% CO_2_. The supernatant was harvested for cytokine immunoassay, and the cells were labelled with antibodies specific for CD11b (Ozyme, Saint Cyr, France, cat. no.: BLE101226) and CD11c (Ozyme, cat. no.: BLE117318), or CD11b (Ozyme, cat. no.: BLE101216) and F4/80 (Ozyme, cat. no.: BLE123152), cell surface markers of BMDCs and BMDMs, respectively, after Fc receptor blocking (BD Pharmingen, Claix, France, cat. no.: 553142) to reduce non-specific binding. To evaluate cellular activation, BMDCs and BMDMs were immunostained with anti-IAb-A^b^ (Ozyme, cat. no.: BLE116410) and anti-CD86 (Ozyme, cat. no.: BLE105008) antibodies. In both cases, only live cells were selected by staining with 7-Aminoactinomycin D (7AAD) (negative gating) (BD Biosciences, cat. no.: 559925) and analysed by flow cytometry using BD™ LSR II (BD Biosciences). The proportion of activated cells was quantified using FCS Express V6 (De Novo Software).

### 2.8. Cytokine Immunoassays

Cytokine production was measured in the supernatant of cell cultures utilising Cytometric Bead Array (CBA) (BD Biosciences, cat. no.: 552364), using a Mouse Inflammation Kit against IL-6, IL-12p70, MCP-1, TNF-α, IL-10, and IFN-γ. The results were acquired by flow cytometry on a BD™ LSR II (BD Biosciences) and analysed with FCAP Array Software v3.0 (BD Biosciences, cat.no: 652099).

### 2.9. The NO and ROS Production

The amounts of NO production by BMDMs and BMDCs were assessed by measuring nitrite concentration in the cell culture media by the Griess assay. An amount of 50 µL of cell supernatants was transferred into a 96-well plate, incubated with an equal volume of Sulphanilamide (Sigma, cat. no.: S9251) and N-alpha-naphthyl-ethylenediamine (NED) (Sigma, cat. no.: 222488) solutions, respectively, and was allowed to sit for 10 min in the dark. Then the optical density (OD) of the solution was then measured at 540 nm, using the CLARIOstar^®^ Microplate Reader (BMG Labtech). The approximate concentration of nitrite in samples was determined from a standard curve. The ROS production by BMDMs and BMDCs was determined by the ROS-Glo™ H_2_O_2_ Assay kit (Promega, cat. no.: G8821). The cells were cultured at a 5 × 10^4^ cells/mL concentration in 96-well plates, exposed to AuNPs 24 h. After that cells were stimulated with 2 µg/mL LPS for 18 h. Then, 20 µL of H_2_O_2_ substrate solution was added for 6 h, followed by addition of 100 µL of the ROS-Glo™ detection solution. After this plate was incubated for 20 min at 22 °C, and luminescence was recorded using the CLARIOstar^®^ Microplate Reader. (BMG Labtech).

### 2.10. Metabolic Flux Analysis

Mature BMDCs (at Day 10) were plated at 1.5 × 10^5^ cells per well in the Seahorse culture plate (Agilent, cat. no.: 102416-100) pre-coated with Cell-Tak (Sigma, cat. no.: 354240) in complete culture medium supplemented by GM-CSF (5 ng/mL) and flt3L (25 ng/mL). Mature BMDMs (at Day 7) were plated at 0.8 × 10^5^ cells per well in the Seahorse culture plate. For both cells types, 1 h after plating, the AuNPs were added. After 24 h of culture, they were washed, and stimulated or not with LPS/IL-4. Then after 24 h, they were either washed with glycostress assay medium [XF base medium supplemented (Agilent, cat. no.: 103575-100) with 1 mM glutamine (Agilent, Santa Clara, CA, USA, cat. no.: 103579-100)) or Mito stress assay medium (XF base medium supplemented with 1 mM pyruvate (Agilent, cat. no.: 103578-100), 2 mM glutamine and 10 mM glucose (Agilent, cat. no.: 103577-100)), and then replenished with the same medium (180 µL/well). The cell culture plate was placed into a 37 °C incubator in the absence of CO_2_ for 45 min to 1 h before the assay. The Seahorse XFe96 took analyser takes measurements of the extracellular acidification rate (ECAR) and the oxygen consumption rate (OCR) every 6 to 7 min. During the course of experiment, inhibitors were introduced to determine which metabolic parameters were affected because of the AuNPs treatment by measuring the ECAR and OCR. Mitochondrial metabolism of BMDCs and BMDMs was monitored by the mitostress assay. The inhibitors for mitostress assay, added in the listed order, were Oligomycin (Sigma, cat. no.: 75351) (1.5 µM—inhibits F0/F1 ATPase), Carbonyl cyanide-p-trifluoromethoxyphenylhydrazone (Sigma, cat. no.: C2920) (1.5 µM—uncoupling agent), antimycin A (Sigma, cat. no.: A8674), and rotenone mixture (Sigma, cat. no.: R8875) (1 µM—inhibits mitochondrial respiratory complexes 3 and 1, respectively). Glycolysis of BMDCs and BMDMs was monitored by the glycostress assay. For the glycostress assay glucose (10 mM), Oligomycin (1.5 µM), and 2-deoxy-D-glucose (2-DG) (Sigma, cat. no.: D8375) (30 mM-inhibits glycolysis) were injected sequentially. For each assay, Hoechst 33342 (ThermoFisher, cat. no.: H21492) was injected at the end, to normalise the data based on cell count. A graphical representation of the experimental design is presented in Supplementary Figure S2.

### 2.11. Antigen Presentation Assay

The AuNPs exposed BMDCs were stimulated with 2 µg/mL LPS for 4 h and incubated with 25 µg/mL OVA for another 4 h at 37 °C and 5% CO_2_. A total of 0.4 × 10^6^ T cells (extracted from OT-II mice and resuspended in the culture medium of BMDCs) were added to 0.1 × 10^6^ BMDCs. Co-cultures were incubated for 4 d, and supernatants were then harvested for cytokine immunoassays to measure IFN-γ and IL-17 by using the Th1/Th2/Th17 CBA kit (BD Biosciences, cat. no.: 560485), and IL-13 (ThermoFisher, cat. no.: 88-7137-77) was determined by ELISA. A graphical representation of the experimental design is presented in Supplementary Figure S3.

### 2.12. Statistical Analysis

Results are expressed as mean values ± SD. Statistical analysis was performed using Prism version 8.4.2 (GraphPad). Statistically significant differences were assessed by ordinary one-way ANOVA, or repeated measures (RM) ANOVA with Tukey’s multiple comparisons test. Significance of the results is indicated according to *p*-values was as follows: * *p* ≤ 0.05, ** *p* ≤ 0.01, *** *p* ≤ 0.001 and **** *p* ≤ 0.0001. The *p*-value below 0.05 was considered statistically significant.

## 3. Results

### 3.1. Viability of APCs after Exposure to AuNPs

**Figure 1:**
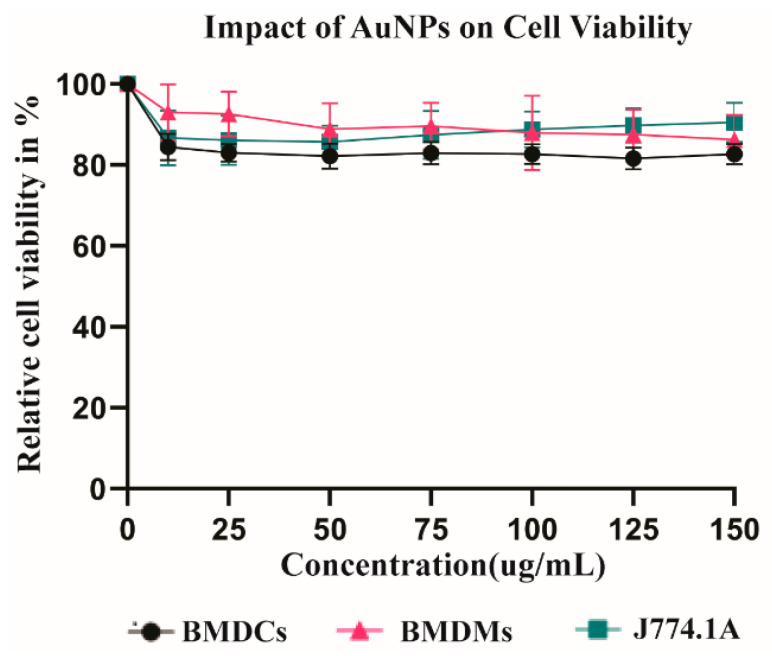
Evaluation of cell toxicity after gold nanoparticles (AuNPs) exposure of antigen-presenting cells (APCs). Cell mortality (lactic dehydrogenase (LDH) Assay) of BMDCs, bone marrow-derived macrophages (BMDMs), and J774.1A cells was analysed after exposure to different concentrations of AuNPs for 24 h. Data are normalised to untreated cells (no AuNPs), considered 100% live. The results are the mean and standard deviation of three independent experiments.

To analyse whether AuNPs are toxic for APCs, dendritic cells (BMDCs) and macrophages (BMDMs and J774.1A) were exposed to different concentrations of AuNPs in vitro. Cell viability was evaluated after 24 h by measuring membrane integrity through the LDH release assay (Figure 1). For concentrations up to 150 µg/mL, no significant cytotoxicity of AuNPs was observed (Figure 1). For the analysis of the immune cell functions, we later used 50 µg/mL as the highest concentration of AuNPs. This was due to a constraint in some experimental set up, like in the metabolic analysis, limiting the volume of AuNPs that could be added.

### 3.2. The AuNPs Uptake by APCs

**Figure 2:**
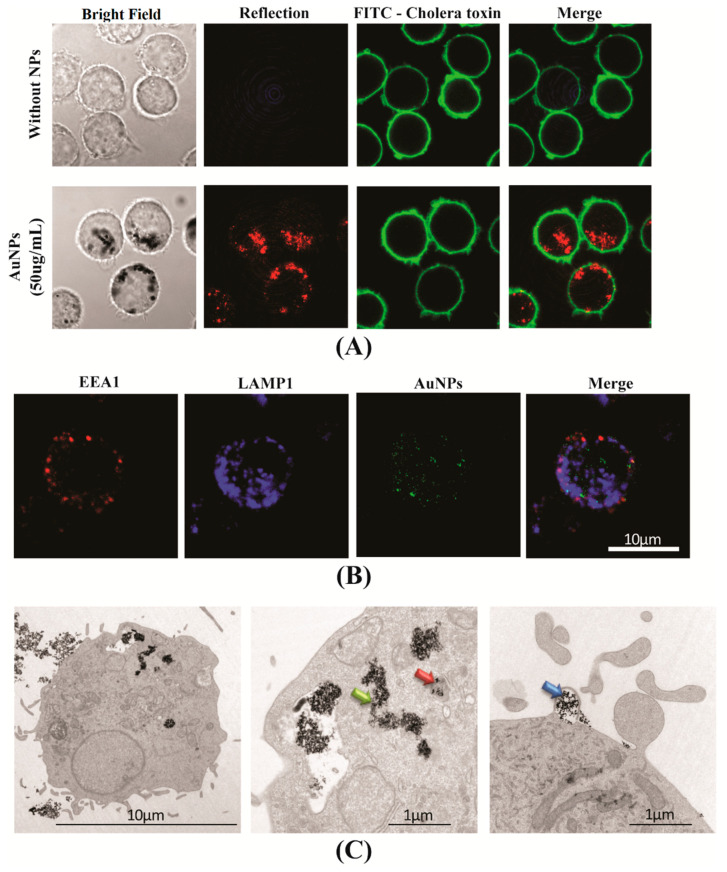
Uptake of AuNPs by APCs. (**A**,**B**) are images of confocal microscopy. (**A**) After exposure of the J774. 1A cells to 50 µg/mL AuNPs for 24 h, cell membranes were labelled with FITC-coupled cholera toxin (green), and AuNPs were observed by the reflection using a 560 nm laser (red). (**B**) the BMDCs were exposed to 75 µg/mL AuNPs for 10 min; early (EEA1) and late (LAMP1) endosomes were labelled and AuNPs were observed by the reflection of 488 nm laser. (**C**) The intracellular location of AuNPs (13 µg/mL) in BMDCs was analysed using transmission electron microscopy after a 18-h incubation. The central and right panels represent magnified views of cells. Images are representative of the majority of cells observed.

To visualise the internalisation of AuNPs by APCs, we first used J774.1A cell line as a model of phagocytic cells. Cells were exposed to 50 µg/mL of AuNPs for 24 h and observed by confocal microscopy, AuNPs were visualised by light reflection, cellular plasma membranes were stained using FITC labelled cholera toxin. Figure 2A clearly shows the internalisation of AuNPs into J774.1A cells. To more precisely map the sub-cellular compartments of the internalisation, primary phagocytic cells, BMDCs, were exposed to AuNPs for 24 h, then early endosomes and late endosomes were labelled with anti-EEA1 antibodies and anti-LAMP1 antibodies, respectively. Confocal imaging revealed that AuNPs appeared scattered throughout the cell with no specific accumulation with either early or late endosomes (Figure 2B). The intracellular location of AuNPs in BMDCs was further analysed by electron microscopy, confirming some AuNPs were inside intracellular vesicles (orange arrow) and also that AuNPs accumulated in the cytoplasm (green arrow) or associated to the membrane, probably being internalised (blue arrow) (Figure 2C). Accumulation of AuNPs into phagocytic APCs raises the question of whether their functions could be altered.

### 3.3. Phagocytosis Capacity of Macrophages

**Figure 3:**
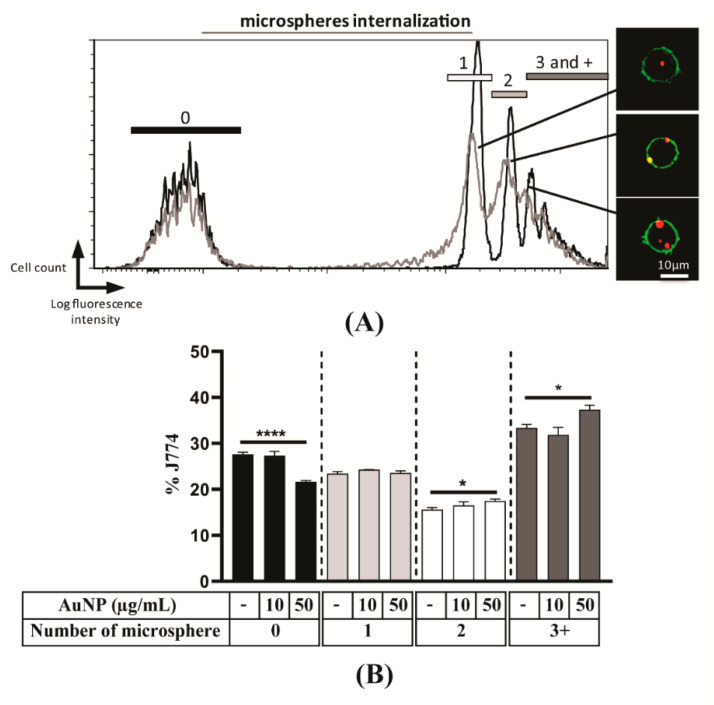
Phagocytosis capacity of macrophages. (**A**) J774.1A were exposed (grey) or not (black) to 50 µg/mL AuNPs for 24 h and incubated with fluorescent microspheres Fluospheres^®^ for 6 h, and analysed by flow cytometry. Overlaid histograms are shown. (**B**) After exposure to different concentrations of AuNPs, the proportion of cells in each peak was analysed. The results are the mean +/− SD of three independent experiments. Ordinary one-way ANOVA was performed * *p* ≤ 0.05, **** *p* ≤ 0.0001.

The J774.1A Ms. are a commonly used model to analyse internalisation processes and phagocytosis. The phagocytic capacity of J774.1A Ms. was evaluated via engulfment of polystyrene microspheres (1 µm) coupled with a fluorochrome. After sorting, the number of phagocytosed polystyrene microspheres was counted by confocal microscopy. Different sub-populations could be defined depending on their fluorescence intensity. Furthermore, fluorescence intensity can be correlated to a number of phagocytosed polystyrene microspheres counted by confocal microscopy (Figure 3A). Thus, each peak of the flow cytometry histogram has been assigned to a cell population which has internalised 1, 2, 3, and more microspheres respectively. After exposure to AuNPs, the same analysis was performed (Figure 3B) and showed a moderate increase of phagocytosis at the highest dose of AuNPs that is reflected by a decrease of the number of cells, which did not contain microspheres. In conclusion, the exposure of macrophages to AuNPs does not lead to important change in their subsequent phagocytosis capacity.

### 3.4. The AuNPs Do Not Impact the Expression of Cell Surface Markers of APCs Activation

**Figure 4:**
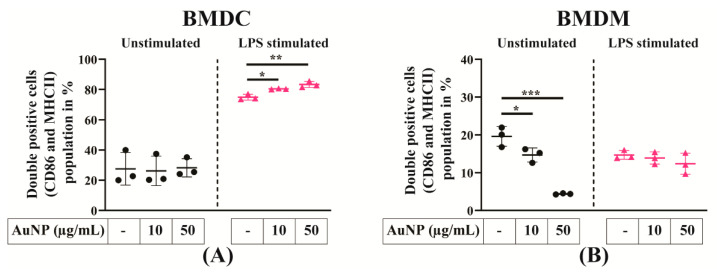
Expression of activation surface markers of APCs. (**A**) Expression of activation markers of BMDCs. (**B**) Expression of activation markers of BMDMs. APCs are exposed to AuNPs for 24 h, followed by lipopolysaccharide (LPS) stimulation for an additional 24 h. Percentage of double-positive (CD86 and class II major histocompatibility complex (MHC-II)) BMDCs and BMDMs are counted gated on CD11b and Cd11c positive cells for BMDCs and CD11b and F4/80 positive cells for BMDMs and plotted in a bar graph. The results are mean +/− SD of three independent experiments. Ordinary one-way ANOVA was performed * *p* ≤ 0.05, ** *p* ≤ 0.01 and *** *p* ≤ 0.001.

The impact of AuNPs on APCs cell activation was analysed using primary BMDCs and BMDMs; being not transformed these cells better reproduce cellular fate than cell lines. After the differentiation of BM cells in the culture, BMDCs and BMDMs were analysed by flow cytometry for the expression of differentiation surface markers. The BMDCs were stained for CD11b and CD11c expressions and BMDMs for CD11b and F4/80 expressions (Supplementary Figure S4). For each cell preparation, live cells were gated by selecting the 7AAD negative cell population, and double-positive cells were gated to analyse the expression of the activation markers CD86 and MHC-II (Figure 4A,B). As expected after the activation of APCs by LPS, the number of activated cells expressing both CD86 and MHC-II increased from 27.57% to 74.97% for BMDCs (Figure 4A), but a negligible change was observed in the case of BMDMs (18.43 to 14.69%) (Figure 4B). Although LPS did not alter the double- positive cell population in BMDMs, however, in case of BMDMs, CD86 expression significantly increased (43.25 vs. 84.03%) (Supplementary Figure S4). Exposure to increasing concentrations of AuNPs did not yield to the activation of BMDCs, the level of double-positive cells remaining in the same range between 27.57% to 28.26%, but decreased in the case of BMDMs (18.43% vs. 4.39%). These data led us to conclude that AuNPs cannot activate BMDCs but can suppress BMDMs activation. When cells were exposed to AuNPs, their capacity to respond to LPS activation increased for BMDCs (74.97% vs. 83.37%), but remain unchanged for BMDMs (14.69% vs. 12.37%). The AuNPs-mediated modulation of cell surface marker expression is presented in Table 2, and the analysis of flow cytometry data is shown in Supplementary Figure S4. Altogether, these data indicate that exposure to AuNPs affect APCs activation estimated by the expression of cell surface markers.

### 3.5. The AuNPs Do Not Modify the Secretions of Signalling Factors by APCs

**Figure 5:**
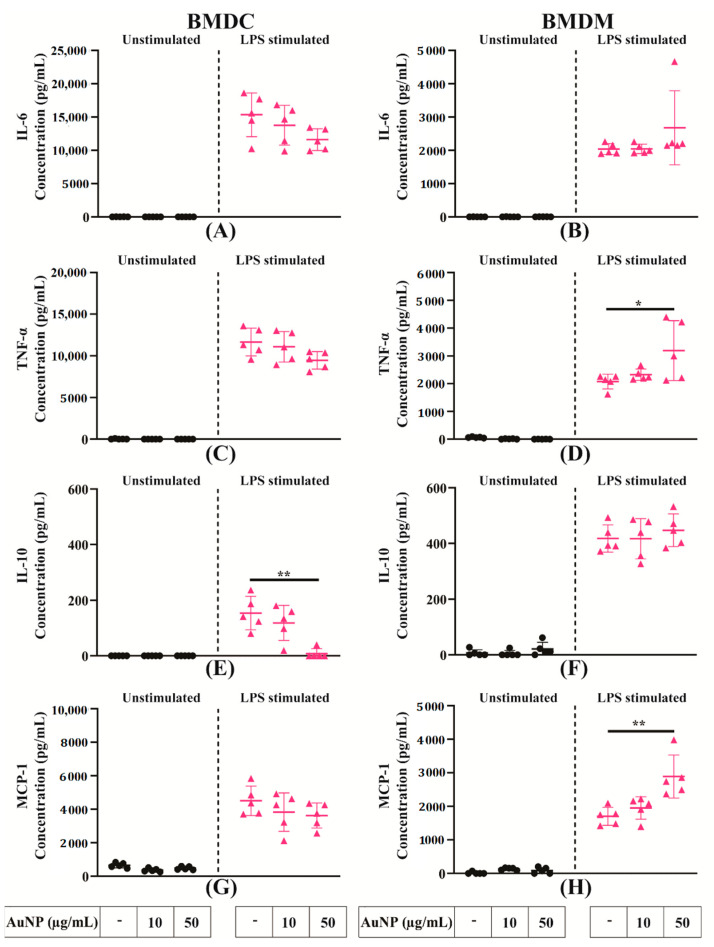
Secretion of different signalling factors by activated APCs. (**A**–**H**) Relative cytokine and chemokine concentrations in the supernatant of BMDCs and BMDMs exposed to AuNPs and activated by LPS. Results are mean +/− SD of five independent experiments. Ordinary one-way ANOVA was performed * *p* ≤ 0.05 and ** *p* ≤ 0.01.

The production of soluble factors, including signalling proteins like cytokines or chemokines and small molecular mediators like NO and ROS, are important features of APCs responses to activation. To determine whether the exposure of APCs to AuNPs could modulate their capacity to secrete cytokines and chemokines, supernatants of BMDCs and BMDMs cultures were collected and quantified for their content in pro-inflammatory (IL-6, TNF-α), immunoregulatory cytokines (IL-10, IL-12) and chemokine (MCP-1). The AuNPs by themselves did not induce any cytokine and chemokine secretion (Figure 5). As expected, in response to LPS activation, both BMDCs and BMDMs secreted cytokines and chemokines, and their exposure to AuNPs had little impact on their cytokine secretion capacity to APCs. The production of IL-10 by BMDCs reduced at the highest concentration of AuNPs. The BMDMs showed a little but significant increase of TNF-α, and of MCP-1 only at the highest concentration of AuNPs. In conclusion, AuNPs exposure has little, if any, effect on the capacity of APCs to secrete cytokines or chemokines in response to inflammatory signalling by LPS.

**Figure 6:**
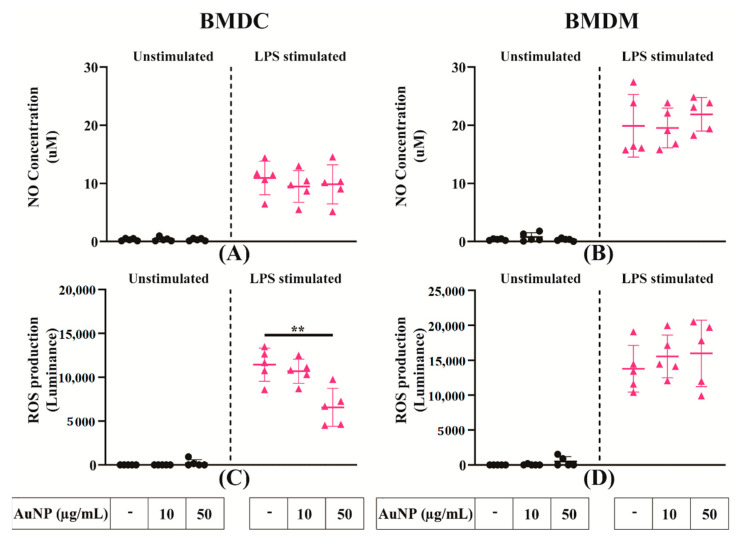
Secretion of different signalling factors by activated APCs. (**A**,**B**) Relative nitric oxide (NO) concentration in the supernatant of BMDCs and BMDMs exposed to AuNPs and activated by LPS. (**C**,**D**) Relative reactive oxygen species (ROS) production by BMDCs and BMDMs exposed to AuNPs and activated by LPS. Results are mean +/− SD of five independent experiments. Ordinary one-way ANOVA was performed ** *p* ≤ 0.01.

The NO and ROS productions were evaluated in the culture supernatant of APCs cultures with the Griess assay and the quantification of H_2_O_2_, respectively. No indication of NO or ROS production was obtained after exposure of both APCs to AuNPs (Figure 6A–C). After stimulation of LPS, both APCs produced significant quantities of these mediators. In the case of BMDMs, these productions were not modified by exposure to AuNPs (Figure 6B,D); BMDCs showed a moderate reduction of ROS productions (Figure 6C) with NO production remaining unchanged (Figure 6D). These data indicate that BMDCs and BMDMs are differently affected by AuNPs in their capacity to produce NO and ROS after LPS activation.

### 3.6. Analysis of the Mitochondrial Metabolism of APCs

Cellular metabolism is of particular interest, considering its implication in many cell functions. Under the influence of their micro-environment, APCs are polarised to achieve distinct functions. For instance, when APCs are challenged with LPS, they polarise towards pro-inflammatory M1 phenotype; conversely, when they are stimulated with IL-4, they develop an anti-inflammatory M2 phenotype. Moreover, it is known that the metabolism of LPS-activated BMDCs and BMDMs relies mainly on glycolysis, whereas cells activated by IL-4 are dependent on mitochondrial metabolism.

**Figure 7:**
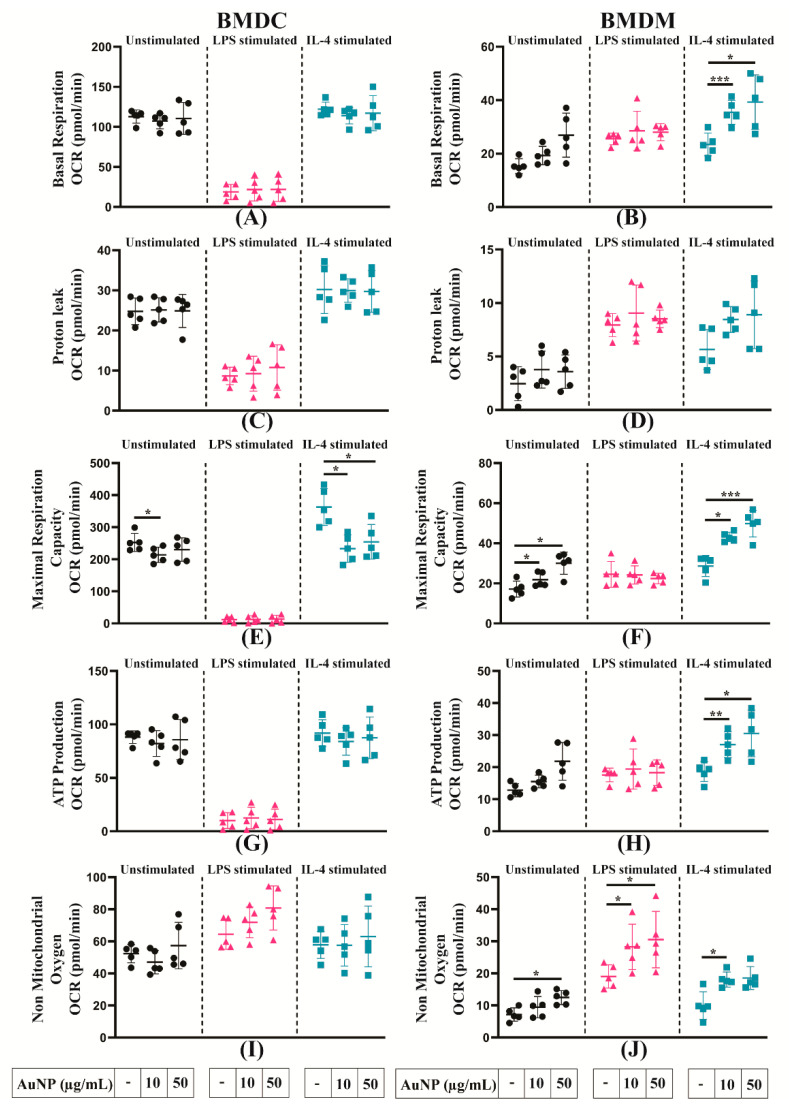
Mitochondrial metabolism of APCs activated or not. (**A**,**B**) Basal respiration, (**C**,**D**) H+ (Proton) leakage, (**E**,**F**) maximal respiration, (**G**,**H**) ATP production, (**I**,**J**) non-mitochondrial respiration of BMDCs and BMDMs was measured after a 24 h exposure to AuNPs or not stimulated by LPS or IL-4 for another 24 h or not, as indicated in the graphs below. After measuring the oxygen consumption rate (OCR) by using the Seahorse XFe96 analyser, data were normalised based on the cell number by using Hoechst 33342 staining. The results are mean +/− SD from five independent experiments. RM one-way ANOVA was performed * *p* ≤ 0.05, ** *p* ≤ 0.01 and *** *p* ≤ 0.001.

To reveal the impact of AuNPs on mitochondrial metabolism of differently polarised cells, the cells were pre-treated with different concentrations of AuNPs, then stimulated with LPS or IL-4 or unstimulated for 24 h. The OCR was measured by the Seahorse XFe96 analyser. As shown in Figure 7A, AuNPs exposure did not induce any alteration of the basal mitochondrial respiratory capacity of BMDCs, activated by LPS or IL-4 or not. Similarly, pre-treatment of AuNPs had no significant effect on the basal respiratory capacity of unstimulated or LPS-stimulated BMDMs; however, IL-4-stimulated BMDMs showed a significant increase of their basal respiration in a concentration-dependent manner (Figure 7B). The AuNPs did not alter proton leakage for BMDCs and BMDMs in the stimulated or unstimulated states (Figure 7C,D).

The maximal respiration capacity decreased after AuNPs exposure of unstimulated BMDCs and, more importantly, in IL-4-stimulated BMDCs (Figure 7E). Of note, LPS stimulation led to a complete abolishment of the maximal respiratory capacity of BMDCs. The behaviour of BMDMs was different, a significant increase in the maximal respiration capacity of unstimulated and IL-4-stimulated BMDMs was observed, while no change was observed in LPS-stimulated BMDMs (Figure 7F). The analysis of ATP production (Figure 7G,H) showed similar profiles as those observed for basal respiration, with no modification after AuNPs exposure of the stimulated or unstimulated BMDCs, but a significant increase in IL-4-stimulated BMDMs, and no alteration in LPS and unstimulated BMDMs. The non-mitochondrial oxygen consumption of BMDCs was not significantly affected whatever their stimulation status (Figure 7I). Again, BMDMs displayed different reactivity, with an increase in the non-mitochondrial respiratory capacity for all types of BMDMs (Figure 7J).

Furthermore, we measured the spare respiratory capacity, which indicates the cell ability to respond to the increasing demand for ATP during the period of stress. Our results revealed that BMDCs spare respiratory capacity follows the same profile as that of maximal respiratory capacity (Supplementary Figure S5A), which is not observed in BMDMs. However, the results are not conclusive because of high variability (Supplementary Figure S5B). The analysis of coupling efficiency showed that AuNPs did not influence the coupling efficiency of stimulated or unstimulated BMDCs (Supplementary Figure S5C) and BMDMs (Supplementary Figure S5D), indicating there was no leakage of protons, which is essential for driving ATP synthase.

Altogether, these data show that the metabolism of BMDCs and BMDMs are differently affected by the exposure to AuNPs, BMDCs are moderately affected, whereas BMDMs significantly increase their mitochondrial and non-mitochondrial respiratory capacities.

### 3.7. Analysis of the Glycolysis of APCs

**Figure 8:**
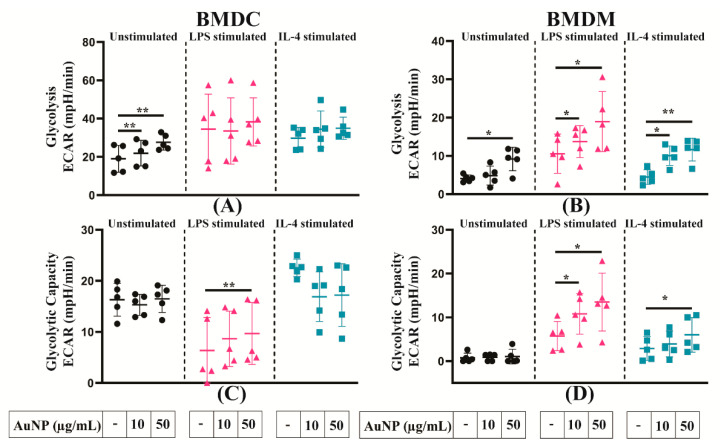
Glycolysis of APCs activated or not. (**A**,**B**) Glycolysis, (**C**,**D**) glycolytic Capacity of BMDCs and BMDMs were evaluated after a 24 h exposure to AuNPs or not and after stimulation or not by LPS or IL-4 for another 24 h, as indicated in the graphs below. After measuring the extracellular acidification rate (ECAR) using the Seahorse XF analyser, the data were normalised based on the cell number by using Hoechst 33342 staining. The results are mean +/− SD of five independent experiments. RM one-way ANOVA was performed * *p* ≤ 0.05 and ** *p* ≤ 0.01.

The effects of AuNPs exposure on the metabolism of APCs were further investigated by the analysis of their glycolytic activity. The BMDMs and BMDCs were pre-treated with different concentrations of AuNPs, then stimulated with LPS or IL-4 or kept unstimulated for 24 h. The extracellular acidification rate (ECAR) was measured by the seahorse XFe96 analyser. As shown in Figure 8A, AuNPs exposure led to a slight increase of glycolysis in unstimulated BMDCs, whereas stimulated BMDCs displayed high variable levels of glycolysis. All types of BMDMs responded to AuNPs exposure by significant increases in glycolysis (Figure 8B). Figure 8C depicted that pre-treatment with AuNPs increased the glycolytic reserve of LPS stimulated BMDCs but not that of unstimulated and IL-4-stimulated BMDCs. An increase was observed in LPS and IL-4-stimulated BMDMs, but unstimulated cells showed no alteration when exposed to different concentrations of AuNPs (Figure 8D). Overall, AuNPs increased glycolysis dependent energy demand in both BMDCs and BMDMs in a manner dependent on the dose, and on the state of stimulation.

### 3.8. The AuNPs Exposure to BMDCs Increases the Antigen Specific the T Cell Responses

**Figure 9:**
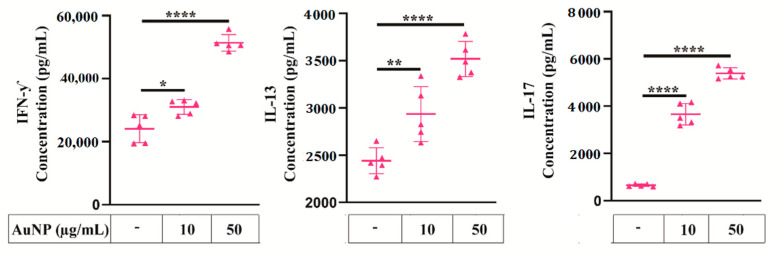
Antigen-specific T-cell responses. [A] OT-II T cells were co-cultured with AuNPs exposed/ovalbumin (OVA) loaded/LPS activated BMDCs for 4 days. T cell cytokine production is shown. In the absence of OVA antigen, the concentrations of IFN-γ, IL-13, and IL-17 produced by T cells are 186, 35, and 623 pg/mL, respectively (data not shown), and in the absence of OVA antigen but the presence of AuNPs, the concentrations of IFN-γ, IL-13, and IL-17 produced by T cells are 90 pg/mL, 24 pg/mL, and 381 pg/mL, respectively (data not shown). The results are mean +/− SD of five independent experiments. Ordinary one-way ANOVA was performed * *p* ≤ 0.05, ** *p* ≤ 0.01 and **** *p* ≤ 0.0001.

To explore whether alterations of the metabolic profile of APCs impair the activation of T cell, we performed an in vitro antigen presentation assay. Briefly, AuNPs exposed BMDCs were activated with LPS and a model antigen OVA, then co-cultured with LT purified from the spleen of OT-II transgenic mice. The OT-II mice exhibit a transgenic T cell receptor (TCR), which recognises OVA peptide presented by APCs on MHC-II IAb; consequently, all LT from these mice are specific to OVA. After 4 days, the supernatant of the co-cultures was collected and the fate of LT was evaluated with regard to the cytokines secreted: gamma interferon (IFN-γ) as the major effector of Th1 response, IL-13 and IL-4 for Th2 response and IL-17 as representative of Th17 response. These cytokine secretions were analysed by the CBA. Figure 9 shows the quantification of these cytokines for a representative experiment. The AuNPs by themselves did not induce cytokine secretion by LT. Additionally, LDH mediated toxicity analysis showed AuNPs did not directly induced T cell death (Supplementary Figure S6). The production of IFN-γ, IL-13, and IL -17 significantly increased when BMDCs were exposed to a high concentration of AuNPs (50 µg/mL). Altogether, these data showed AuNPs exposure increased BMDCs capacity to activate antigen specific responses of all T cells, i.e., Th1, Th2, Th17.

## 4. Discussion

Gold salts have long been used to treat various diseases such as rheumatoid arthritis and tuberculosis [18]. Today, AuNPs are widely used in targeted drug delivery, cell imaging, cancer diagnostics, and treatments [1]. The effectiveness of these particles depends on their ability to target specific cells followed by their internalisation. The nanometric size of AuNPs plays a critical role in their internalisation; 50 nm AuNPs have been shown to be the most efficiently internalised by cells [19]. As these nanoparticles are internalised and accumulated inside cells, they may interfere with the cellular fate and functions. This issue is especially important concerning the immune system, because of the capacity of some cells of this system to actively capture extracellular materials and control immune responses by the delivery of inflammatory signals for stimulation and orientation of antigen presentation, leading to specific immune responses. A precise knowledge of the effects of AuNPs on APCs, including macrophages (Ms) and dendritic cells (DCs), provides a valuable insight into the biological consequences of AuNPs exposures and to define putative adverse effects. It is yet well established that NPs immunotoxicity should be tested on APCs [20,21], because these cells are involved in nonspecific innate defences, as well as in specific immune responses. Furthermore, DCs play a critical role as a bridge between innate and adaptive immune systems by initiating the T cell-mediated response [22]. Therefore, perturbation of the functions of these cells may result in altered immune response in NP-exposed individuals. In consequence, Ms and DCs appear to be appropriate tools for discriminating between NPs that interfere with the immune system or not. The current work provides a comparative study of the influence of AuNPs at subtoxic concentration on two primary professional phagocytic cells derived from the bone marrow of mice, BMDCs and BMDMs. Their activities and characteristics: phagocytosis, cell activation, cytokine secretions, redox status together with their ability to present antigens was analysed in detail. In addition, we provide a study of the metabolic activity, pointing at different influences of AuNPs on mitochondrial metabolism and glycolysis of BMDCs and BMDMs.

The phagocytic capacity of APCs leads to the accumulation of NP inside cells, therefore it is important to investigate the sub cellular localisation of AuNPs in APCs. Accumulation of AuNPs inside intracellular vesicles of the J774.A1 Ms cell line [23] and, more precisely, in the lysosome of the raw 264.7 Ms cell line [24], has been shown by TEM and confocal microscopy. In the present study, we demonstrate the presence of AuNPs in early (EEA1+) and late (LAMP1+) endosomes of APCs by confocal microscopy. In addition to AuNPs identified in EEA1 and LAMP1 vesicles, aggregated clusters of AuNPs were observed in the cytosol of APCs using confocal microscopy and more precisely with TEM.

One of the key properties of APCs is their ability to engulf foreign material by phagocytosis. The exposure to 10 nm AuNPs was shown to significantly reduce the phagocytic capacity of Raw 264.7 Ms cell line and BMDMs [25]. In the present study, we show that the phagocytic capacity of the J774.1A Ms cell line is not impaired after 24 h exposure with AuNPs. A possible explanation for these different effects could be that different cell lines of Ms were used and/or related to the nature of the phagocytosis assay that is based on the use of *E. coli* [25] and of polystyrene beads in our study.

Accumulation of nanoparticle inside the APCs could be an important perturbation for the activation state of the cells. Cell interaction with nanoparticle may hinder their activation state which could be evaluated by the cell surface expression of CD86 and MHC-II. We found that AuNPs did not activate the BMDCs by themselves but increase the response of BMDCs to LPS. This finding is consistent with previous studies showing the increased expression of MHC-II [14] and CD86 [26]. Interestingly, BMDMs display a different behaviour: treatment with AuNPs mildly reduced the expression of activation markers in a dose dependent manner. However, AuNPs treated BMDMs did not show any alteration of the expression of activation markers upon LPS stimulation, which may be due to the saturation of CD86 and MHC-II expression. However, in the case of BMDMs an increase of CD86 expression upon LPS stimulation is more evident than both the CD86 and MHC-II expression level.

Upon activation, APCs secrete several immune-regulatory molecules. We showed that AuNPs by themselves did not fuel any cytokine secretion. These data are in the direct line of previous studies on DCs [14] and on Ms [25], indicating that AuNPs did not promote production of TNF-α, IL-6 and IL-10. These findings were also validated by other previous publications in various models [24,27]. However, we see a mild increase in TNF-α and MCP-1 production in AuNPs-treated BMDMs upon LPS stimulation at a high concentration (50 μg/mL).

We observed that AuNPs neither induced NO and ROS production in unstimulated BMDMs nor altered their production after LPS stimulation. This is consistent with previous findings [27]. Concerning BMDCs, we have seen a significant reduction of ROS production after LPS activation but only at high concentration of AuNPs, while NO production remains unchanged.

Generally, NMs that do not induce inflammatory response are considered as safe. However, there are evidences that NMs might alter the function of immune cells by inducing alterations in metabolic pathways [28,29]. To evaluate the effect of AuNPs on the metabolism of BMDMs and BMDCs, we stimulated them either with either LPS or IL-4 as representatives of different microenvironments, which may affect cellular functions. For example, conventional activation of pro-inflammatory cells by LPS facilitate inflammation and participate to the host defence against various kinds of microbial threats. On the other hand, alternative activation by IL4 induced anti-inflammatory cells, potent suppressors and controllers of ongoing immune responses. Such stimulated cells exhibit a distinguishable regulation of their metabolism: LPS-activated proinflammatory cells undergoing a metabolic switch to enhance glycolysis [30,31]. Alternatively, IL-4 stimulated cells rely on both fatty acid oxidation (FAO) and mitochondrial oxidative phosphorylation (OXPHOS) for sustained energy [10]. Thus, altered metabolism is not only a key feature of stimulated cell function but also a prerequisite for a proper response to immune stimuli. The analysis of mitochondrial metabolism and glycolysis showed that AuNPs did not to alter the basal mitochondrial respiration of BMDCs but increased it for BMDMs. The possible explanation for this phenomenon is the different phagocytic capacity of these cells. Indeed, it has been established that the phagocytic index of Ms is higher than that of DCs [32], thereby enabling accumulation of a large quantities of AuNPs in BMDMs, and increasing the basal respiration to meet the endogenous ATP demand of the cell. Unaltered proton leakage in BMDCs and BMDMs suggests that AuNPs did not induce any mitochondrial damage in these cells. Measurement of ATP production shows that pre-treatment with AuNPs did not alter the ATP production of BMDCs but increased it in BMDMs, which is consistent with basal respiration.

The basal respiration rate does not accurately reflect the ability of cellular respiration to respond to increased energy demand. As such, estimating the maximum capacity of substrate oxidation can be extremely valuable for the discovery of mechanisms by which AuNPs could affect cell metabolism.

Conversely, spare respiratory capacity indicates the reserve capacity of a cell to respond to an increased ATP demand and withstand periods of stress. Here, we show that pre-treatment with AuNPs significantly decreases the maximal and spare respiratory capacities of unstimulated and IL-4-stimulated BMDCs (cells, largely dependent on mitochondrial metabolism), but increases them significantly in unstimulated and IL-4-stimulated BMDMs. The increase of these two parameters in BMDMs can be explained by the increase in basal respiration as well as ATP production due to the increase in cellular energy demand. However, a significant decrease of both these parameters in BMDCs shows the impact of AuNPs on BMDCs. The possible explanation for this could be either alteration of the mitochondrial content and cristae density or alteration of the respiratory substrate transport system or alteration of the respiratory chain complex activity or a combination of these parameters [33]. Benjamin et al. have already hypothesised that AuNPs dependent alteration of innate immune memory in human monocytes might be explained by alterations in metabolic pathways [34]. Here, we confirm that AuNPs alter the metabolic response of BMDMs in response to secondary stimuli (LPS/IL-4).

To be activated, T cells have to recognise the antigen presented by MHC-II molecules and to be stimulated by CD86 accessory molecules, which are both expressed at the surface of DCs, in the context of an inflammatory signal. We observed that BMDCs challenged by LPS as inflammatory signal showed an increased expression of CD86 and MHC-II molecules when they have been exposed to AuNPs. We investigated the impact of BMDCs on T cell antigen responses by the analysis of the secreted cytokines. A significant increase of IFN-γ, IL-13, and IL-17 productions, reflecting Th1, Th2, and Th17 cell polarisation, could be correlated with the activation of the BMDCs seen by high CD86 and MHC-II expression levels. A study with 50 nm AuNPs reported the induction of a Th17 response in humans, but no significant change in Th1 and Th2 responses [35]. This difference can be explained by the cellular model, the surface coating of AuNPs, the maturation, and activation states of DCs and the routes of antigen uptake. Interestingly, the fact that increase T cell responses to antigen affects Th1, Th2, and Th17 subsets of T cells may preserve the balance between pro-inflammatory and anti-inflammatory responses.

In conclusion, our study shows that Ms and DCs respond differently when exposed to AuNPs. Therefore, it is mandatory to take both cell types into account when conducting immunotoxicity testing.

## Figures and Tables

**Table 1:** Concentration of granulocyte-macrophage colony-stimulating factor (GM-CSF), FLT-3L, and IL-6 for bone marrow (BM) derived dendritic cells (BMDCs) culture ^1^.

Cells Are Cultured						
		Day 0	Day 3	Day 5	Day 7	Day 10
Cell Concentration		0.6 × 10^6^/mL	0.5 × 10^6^/mL	0.5 × 10^6^/mL	0.5 × 10^6^/mL	According to Cell Plating
**Supplement**	IL-6	5 ng/mL	2.5 ng/mL	2.5 ng/mL	-	-
	FLT-3	50 ng/mL	40 ng/mL	30 ng/mL	25 ng/mL	25 ng/mL
	GM-CSF	5 ng/mL	5 ng/mL	5 ng/mL	5 ng/mL	5 ng/mL

^1^ BMDCs culture: BMDCs were cultured in a 100 mm TC-treated cell culture dish with 15 mL culture media. Varying concentrations of GM-CSF, FLT-3L and IL-6 were added to on day 0, day 3, Day 5, Day 7, and Day 10 to harvest fully differentiated BMDCs in day 11.

**Table 2:** Percentage of activated APCs with or without AuNPs treatment ^1^.

	BMDCs		BMDMs	
	Unstimulated	LPS Stimulated	Unstimulated	LPS Stimulated
Cells	27.57 ± 8.80	74.97 ± 1.54	18.43 ± 1.64	14.69 ± 0.93
Cells + AuNP_10 μg/mL_	26.21 ± 7.95	80.46 ± 0.23	14.68 ± 1.50	13.88 ± 1.31
Cells + AuNP_50 μg/mL_	28.26 ± 4.95	83.37 ± 1.65	4.39 ± 0.11	12.37 ± 2.25

^1^ Expression of activation surface marker of APCs. Expression of activation marker of BMDCs and BMDMs after exposure to AuNPs for 24 h, followed by LPS stimulation for an additional 24 h. Percentage of double-positive (CD86 and MHC-II) BMDCs and BMDMs were gated on CD11b and Cd11c positive cells for BMDCs and CD11b and F4/80 positive cells for BMDMs and the data is displayed in tabular form. Results are mean +/− SD of 3 independent experiments.

## Data Availability

Not applicable.

## Supplementary Materials

The following are available online at https://www.mdpi.com/2073-4409/10/1/96/s1, Table S1: Hydrodynamic diameter of AuNPs, Figure S1: Experimental scheme of AuNPs, Figure S2: Experimental scheme for metabolic flux analysis, Figure S3: Schematic representation of Antigen presentation assay, Figure S4: Expression of activation surface marker of APC, Figure S5: Effect of AuNPs on Spare respiratory capacity and Coupling Efficiency (%) of activated or un activated BMDMs and BMDCs, Figure S6: Evaluation of cell toxicity after AuNPs exposure of T cells.

## References

[1] Ashraf S., Pelaz B., del Pino P., Carril M., Escudero A., Parak W.J., Soliman M.G., Zhang Q., Carrillo-Carrion C. (2016). Gold-Based Nanomaterials for Applications in Nanomedicine. Top. Curr. Chem..

[2] Khlebtsov B., Panfilova E., Khanadeev V., Bibikova O., Terentyuk G., Ivanov A., Rumyantseva V., Shilov I., Ryabova A., Loshchenov V. (2011). Nanocomposites containing silica-coated gold-silver nanocages and Yb-2,4-dimethoxyhematoporphyrin: Multifunctional capability of IR-luminescence detection, photosensitization, and photothermolysis. ACS Nano.

[3] Farooq M.U., Novosad V., Rozhkova E.A., Wali H., Ali A., Fateh A.A., Neogi P.B., Neogi A., Wang Z. (2018). Gold Nanoparticles-enabled Efficient Dual Delivery of Anticancer Therapeutics to HeLa Cells. Sci. Rep..

[4] Hwang S., Nam J., Jung S., Song J., Doh H., Kim S. (2014). Gold nanoparticle-mediated photothermal therapy: Current status and future perspective. Nanomedicine.

[5] Trombetta E.S., Mellman I. (2005). Cell biology of antigen processing in vitro and in vivo. Annu. Rev. Immunol..

[6] Klopfleisch R. (2016). Macrophage reaction against biomaterials in the mouse model—Phenotypes, functions and markers. Acta Biomater..

[7] Banchereau J., Briere F., Caux C., Davoust J., Lebecque S., Liu Y.J., Pulendran B., Palucka K. (2000). Immunobiology of dendritic cells. Annu. Rev. Immunol..

[8] Blanco P., Palucka A.K., Pascual V., Banchereau J. (2008). Dendritic cells and cytokines in human inflammatory and autoimmune diseases. Cytokine Growth Factor Rev..

[9] Takeda K., Akira S. (2005). Toll-like receptors in innate immunity. Int. Immunol..

[10] O’Neill L.A., Kishton R.J., Rathmell J. (2016). A guide to immunometabolism for immunologists. Nat. Rev. Immunol..

[11] Hussain S., Vanoirbeek J.A., Hoet P.H. (2012). Interactions of nanomaterials with the immune system. Wiley Interdiscip. Rev. Nanomed. Nanobiotechnol..

[12] Alkilany A.M., Murphy C.J. (2010). Toxicity and cellular uptake of gold nanoparticles: What we have learned so far?. J. Nanopart. Res..

[13] Rambanapasi C., Zeevaart J.R., Buntting H., Bester C., Kotze D., Hayeshi R., Grobler A. (2016). Bioaccumulation and Subchronic Toxicity of 14 nm Gold Nanoparticles in Rats. Molecules.

[14] Villiers C., Freitas H., Couderc R., Villiers M.B., Marche P. (2010). Analysis of the toxicity of gold nano particles on the immune system: Effect on dendritic cell functions. J. Nanopart. Res..

[15] Fratoddi I., Venditti I., Cametti C., Russo M.V. (2015). How toxic are gold nanoparticles? The state-of-the-art. Nano Res..

[16] Faure M., Villiers C.L., Marche P.N. (2004). Normal differentiation and functions of mouse dendritic cells derived from RAG-deficient bone marrow progenitors. Cell Immunol..

[17] Chen J., Ellert-Miklaszewska A., Garofalo S., Dey A.K., Tang J., Jiang Y., Clément F., Marche P.N., Liu X., Kaminska B. (2021). Synthesis and use of an amphiphilic dendrimer for siRNA delivery into primary immune cells. Nat. Protoc..

[18] Norn S., Permin H., Kruse P.R., Kruse E. (2011). History of gold—with danish contribution to tuberculosis and rheumatoid arthritis. Dan. Med. Arbog.

[19] Chithrani B.D., Ghazani A.A., Chan W.C. (2006). Determining the size and shape dependence of gold nanoparticle uptake into mammalian cells. Nano Lett..

[20] Dobrovolskaia M.A., Shurin M., Shvedova A.A. (2016). Current understanding of interactions between nanoparticles and the immune system. Toxicol. Appl. Pharmacol..

[21] Fröhlich E. (2015). Value of phagocyte function screening for immunotoxicity of nanoparticles in vivo. Int. J. Nanomed..

[22] Tai Y., Wang Q., Korner H., Zhang L., Wei W. (2018). Molecular Mechanisms of T Cells Activation by Dendritic Cells in Autoimmune Diseases. Front. Pharmacol..

[23] Walkey C.D., Olsen J.B., Guo H., Emili A., Chan W.C. (2012). Nanoparticle size and surface chemistry determine serum protein adsorption and macrophage uptake. J. Am. Chem. Soc..

[24] Shukla R., Bansal V., Chaudhary M., Basu A., Bhonde R.R., Sastry M. (2005). Biocompatibility of gold nanoparticles and their endocytotic fate inside the cellular compartment: A microscopic overview. Langmuir.

[25] Bancos S., Stevens D.L., Tyner K.M. (2015). Effect of silica and gold nanoparticles on macrophage proliferation, activation markers, cytokine production, and phagocytosis in vitro. Int. J. Nanomed..

[26] Ye F., Vallhov H., Qin J., Daskalaki E., Sugunan A., Toprak M.S., Fornara A., Gabrielsson S., Scheynius A., Muhammed M. (2011). Synthesis of high aspect ratio gold nanorods and their effects on human antigen presenting dendritic cells. Int. J. Nanotechnol..

[27] Zhang Q., Hitchins V.M., Schrand A.M., Hussain S.M., Goering P.L. (2011). Uptake of gold nanoparticles in murine macrophage cells without cytotoxicity or production of pro-inflammatory mediators. Nanotoxicology.

[28] Dalzon B., Torres A., Diemer H., Ravanel S., Collin-Faure V., Pernet-Gallay K., Jouneau P.-H., Bourguignon J., Cianférani S., Carrière M. (2019). How reversible are the effects of silver nanoparticles on macrophages? A proteomic-instructed view. Environ. Sci. Nano.

[29] Saborano R., Wongpinyochit T., Totten J.D., Johnston B.F., Seib F.P., Duarte I.F. (2017). Metabolic Reprogramming of Macrophages Exposed to Silk, Poly(lactic-co-glycolic acid), and Silica Nanoparticles. Adv. Healthc. Mater..

[30] Everts B., Amiel E., van der Windt G.J., Freitas T.C., Chott R., Yarasheski K.E., Pearce E.L., Pearce E.J. (2012). Commitment to glycolysis sustains survival of NO-producing inflammatory dendritic cells. Blood.

[31] Van den Bossche J., Baardman J., de Winther M.P. (2015). Metabolic Characterization of Polarized M1 and M2 Bone Marrow-derived Macrophages Using Real-time Extracellular Flux Analysis. J. Vis. Exp..

[32] Kiama S.G., Cochand L., Karlsson L., Nicod L.P., Gehr P. (2001). Evaluation of phagocytic activity in human monocyte-derived dendritic cells. J. Aerosol. Med..

[33] Divakaruni A.S., Paradyse A., Ferrick D.A., Murphy A.N., Jastroch M. (2014). Analysis and interpretation of microplate-based oxygen consumption and pH data. Methods Enzymol..

[34] Swartzwelter B.J., Barbero F., Verde A., Mangini M., Pirozzi M., De Luca A.C., Puntes V.F., Leite L.C.C., Italiani P., Boraschi D. (2020). Gold Nanoparticles Modulate BCG-Induced Innate Immune Memory in Human Monocytes by Shifting the Memory Response towards Tolerance. Cells.

[35] Tomic S., Ethokic J., Vasilijic S., Ogrinc N., Rudolf R., Pelicon P., Vucevic D., Milosavljevic P., Jankovic S., Anzel I. (2014). Size-dependent effects of gold nanoparticles uptake on maturation and antitumor functions of human dendritic cells in vitro. PLoS ONE.

