# Supplementary material for: Impact of Gold Nanoparticles on the Functions of Macrophages and Dendritic Cells
Source: Cells. 2021 Jan 7;10(1):96. doi: 10.3390/cells10010096 (PMC7826823; doi:10.3390/cells10010096)
Supplement: Supplementary file 1 [file cells-10-00096-s001.pdf]

Supplementary Table 1. Hydrodynamic diameter of AuNPs

	Water	DMEM
Hydrodynamic diameter	24.43 ± 0.34 nm	97.01 ± 7.29
PdI	0.20 ± 0.01	0.45 ± 0.009

Supplementary Table 1. Hydrodynamic diameter of AuNPs. The hydrodynamic diameter and polydispersity index (PdI) of the AuNPs were measured by dynamic light scattering (DLS) with a 1 µg/mL AuNPs dispersion in complete DMEM media (In presence of 10% FBS) and H_20. Each measurement was performed in three replications at 25°C.

Supplementary Figure 1. Experimental scheme of AuNPs

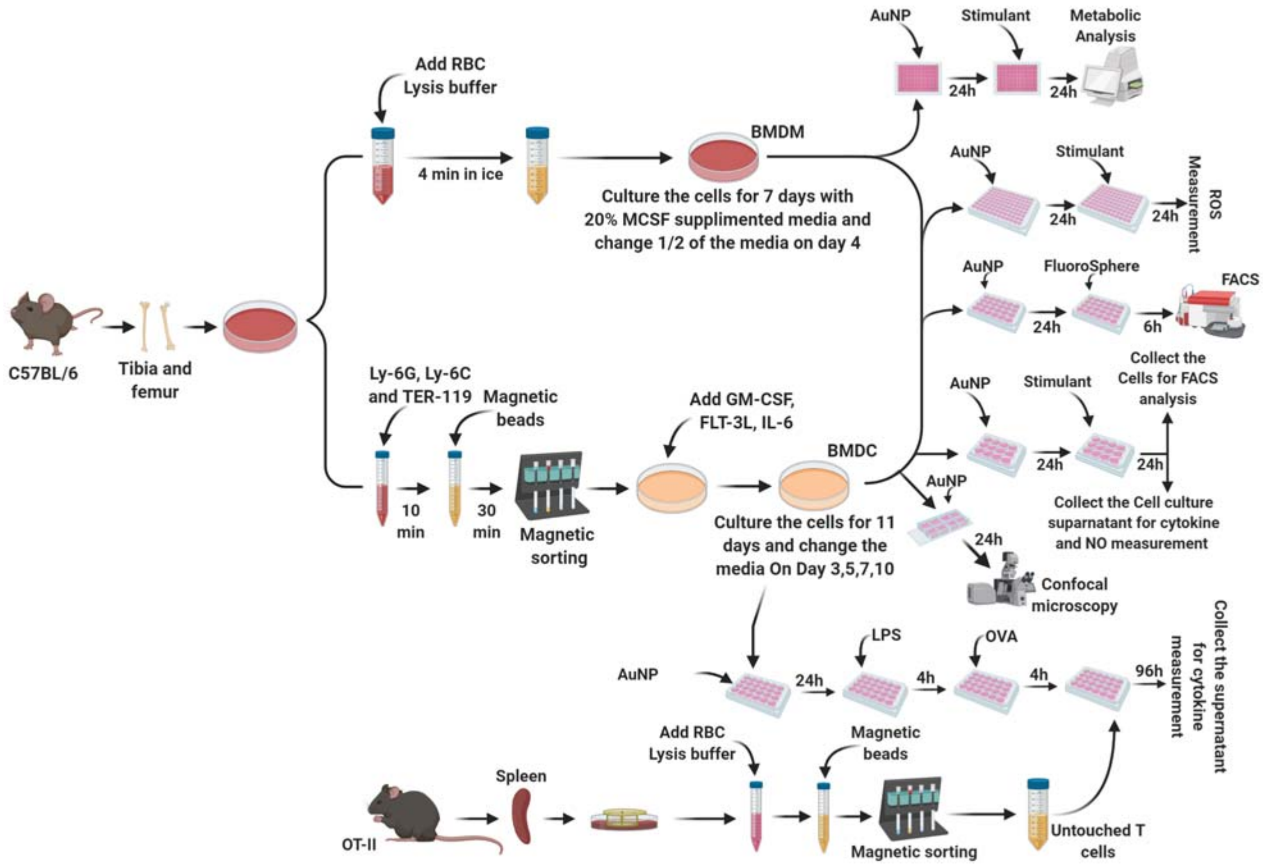

Supplementary Figure 1. Experimental scheme of AuNPs: BMDCs and BMDMs were cultured from mouse bone marrow for 11 and 7 days respectively. After harvesting, the cells were seeded either in 12, 24 or 96 wells plates from Falcon® or Seahorse XFe96 cell culture with AuNPs at 10 and or 50 µg/mL final concentrations. After 24 h of culture, cells were washed and stimulated with LPS or IL-4 for 24 h and downstream experiments were conducted according to the protocol.

Supplementary Figure 2.: Experimental scheme for metabolic flux analysis.

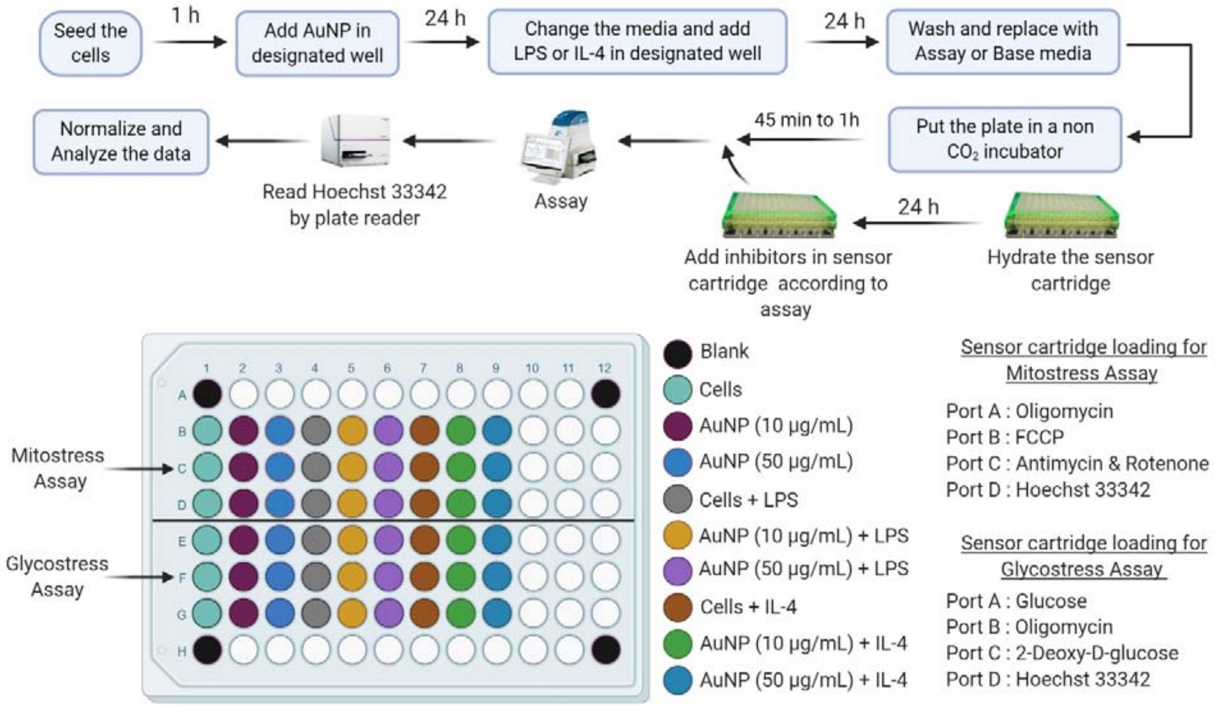

Supplementary Figure 2.: Experimental design of metabolic flux analysis: Mature BMDCs and BMDMs were seeded in Seahorse culture plate. 1 h after plating, cells were treated with AuNPs. After 24 h of culture, cells were washed and left unstimulated or stimulated with LPS/IL-4 for 24 h and the metabolic analysis is done using a Seahorse bio analyzer using the mitostress and glycostress assay protocol.

Supplementary Figure 3. Schematic representation of Antigen presentation assay.

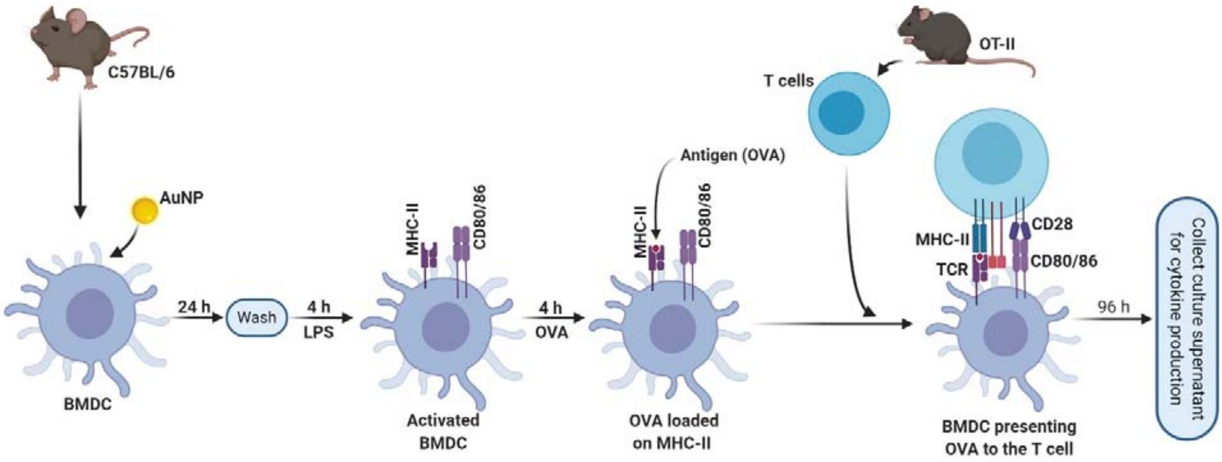

Supplementary Figure 3. Schematic representation of Antigen presentation assay: AuNPs exposed BMDCs were stimulated with 2µg/mL LPS for 4h and incubated with 25µg/mL OVA for additional 4 h at 37°C and 5% CO_2. 0.4 * 10^6 T cells were added to 0.1 * 10^6 BMDCs, at a ratio of 1 BMDCs for 4 T cells. Co-cultures were incubated for 4 days, and then supernatants were harvested for cytokine immunoassays.

Supplementary Figure 4. Expression of activation surface marker of APC.

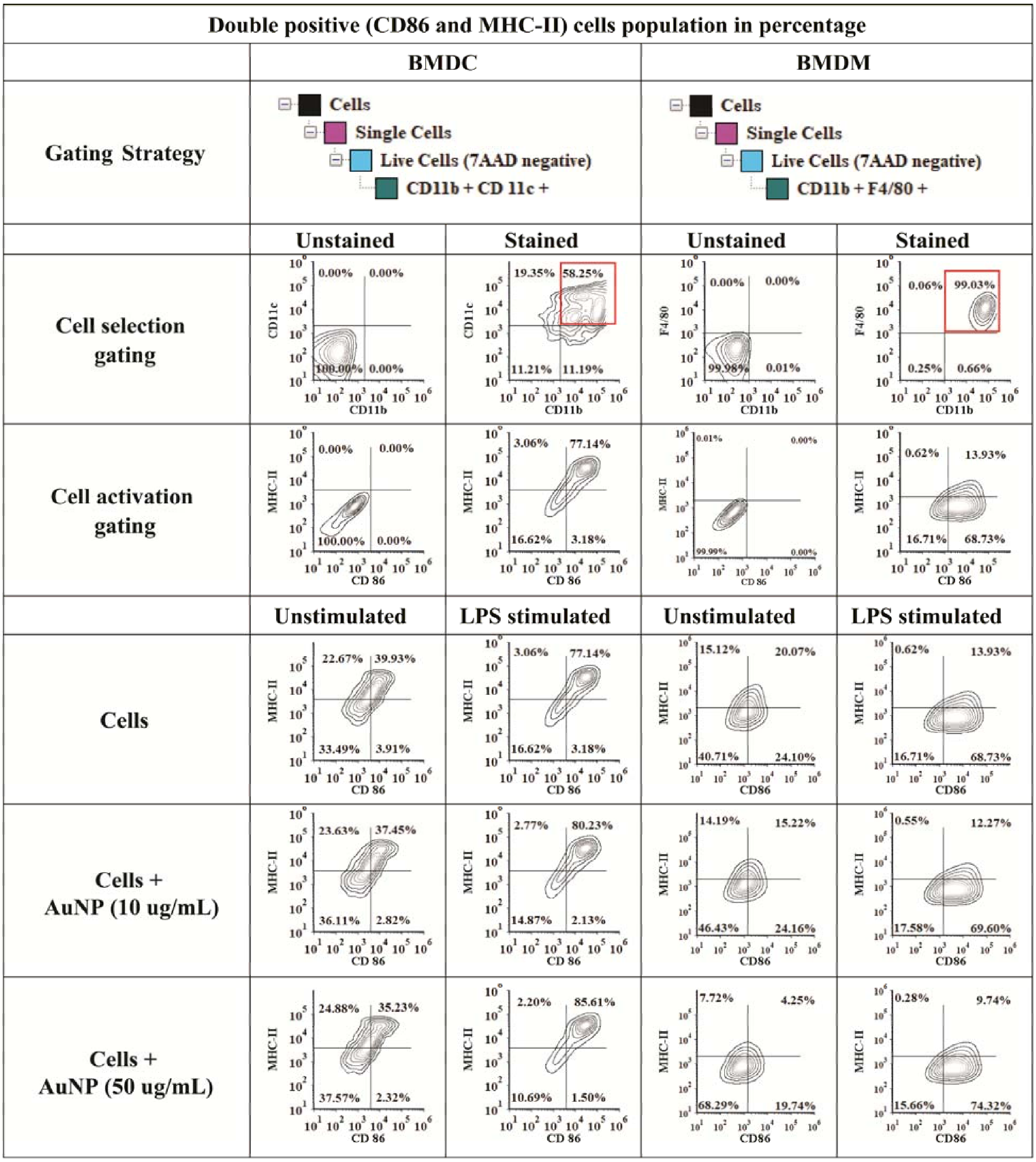

Supplementary Figure 4. Expression of activation marker of BMDCs and BMDMs after exposure to AuNPs for 24 h, followed by LPS stimulation for an additional 24 h. Percentage of double-positive (CD86 and MHC-II) BMDCs and BMDMs were gated on CD11b and Cd11c positive cells for BMDCs and CD11b and F4/80 positive cells for BMDMs and contour graph was displayed. The results are one representative of one of three independent experiments.

Supplementary Figure 5. Effect of AuNPs on Spare respiratory capacity and Coupling Efficiency (%) of activated or un activated BMDMs and BMDCs.

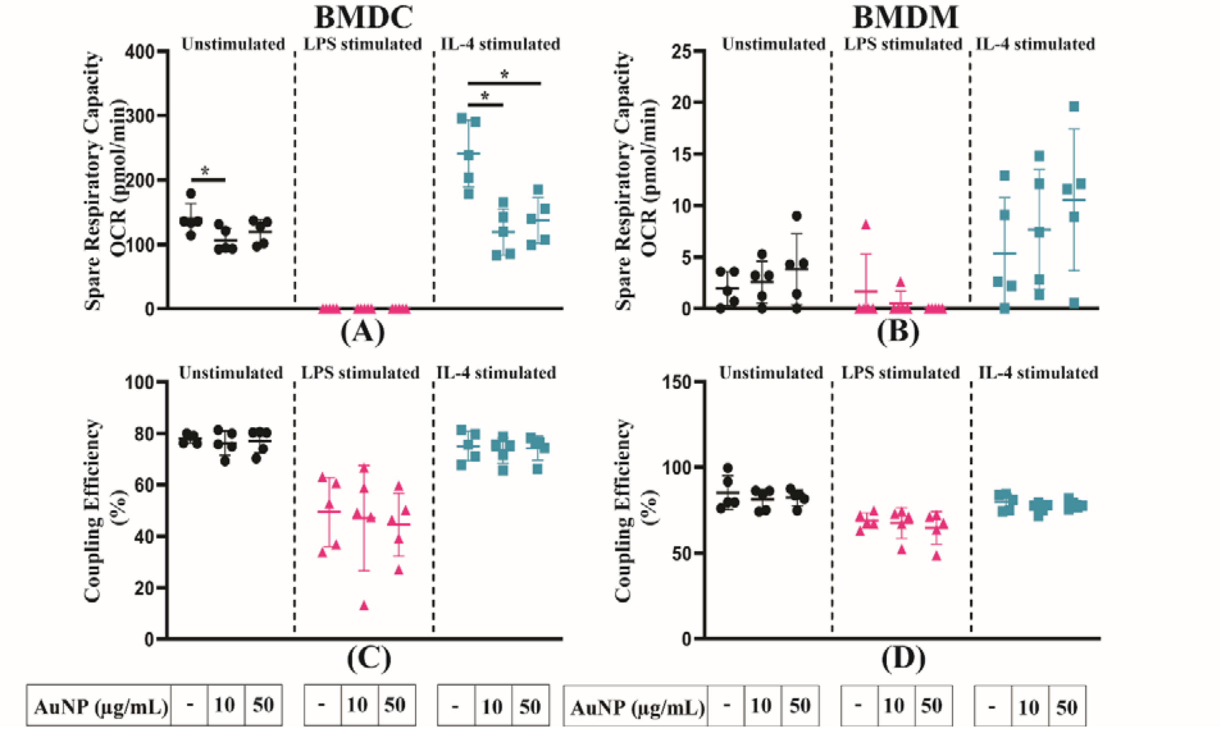

Supplementary Figure 5. Effect of AuNPs on Spare respiratory capacity and Coupling Efficiency (%) of activated or not activated BMDMs and BMDCs. [A, B, C, D] Spare respiratory capacity and coupling efficiency (%). The BMDCs and BMDMs were exposed to AuNPs for 24 h and activated by LPS or IL-4 for another 24 h. After measuring the OCR using the Seahorse XF analyser, data were normalised based on the cell number by using Hoechst 33342 staining. Results are mean +/- SD of 5 independent experiments. RM one-way ANOVA was performed *$p \leq 0.05$.

Supplementary Figure 6. Evaluation of cell toxicity after AuNPs exposure of T cells.

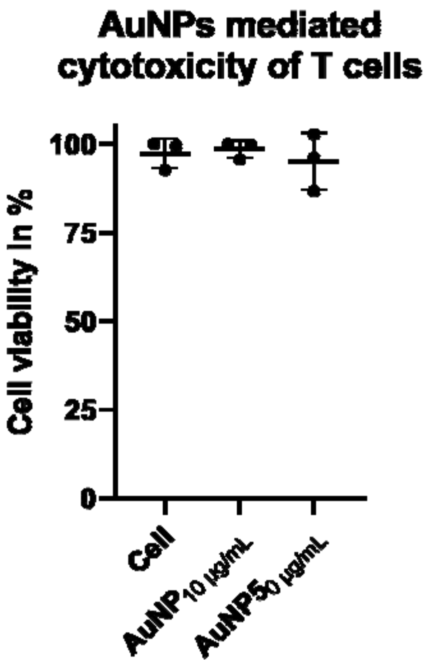

Supplementary Figure 7. Evaluation of cell toxicity after AuNPs exposure of T cells. T cells were extracted from mouse spleen using Dynabeads® Untouched™ Mouse T Cell Kit. Cells were seeded with $CD3^{+}/28^{+}$ beads and either treated with 10 and 50 µg/mL of AuNPs or remain untreated. T cell mortality (LDH Assay) was analysed after 24 h. Results are the mean and standard deviation of 3 independent experiments.
